# Oral microbiomes: more and more importance in oral cavity and whole body

**DOI:** 10.1007/s13238-018-0548-1

**Published:** 2018-05-07

**Authors:** Lu Gao, Tiansong Xu, Gang Huang, Song Jiang, Yan Gu, Feng Chen

**Affiliations:** 1 Central Laboratory, Peking University Hospital of Stomatology, Beijing, 100081 China; 2 Department of Orthodontics, Peking University Hospital of Stomatology, Beijing, 100081 China

**Keywords:** oral microbiomes, human, health, oral diseases, systematic diseases

## Abstract

Microbes appear in every corner of human life, and microbes affect every aspect of human life. The human oral cavity contains a number of different habitats. Synergy and interaction of variable oral microorganisms help human body against invasion of undesirable stimulation outside. However, imbalance of microbial flora contributes to oral diseases and systemic diseases. Oral microbiomes play an important role in the human microbial community and human health. The use of recently developed molecular methods has greatly expanded our knowledge of the composition and function of the oral microbiome in health and disease. Studies in oral microbiomes and their interactions with microbiomes in variable body sites and variable health condition are critical in our cognition of our body and how to make effect on human health improvement.

## Introduction

The discovery of microbes dates back to the 1700s. Historically, Antonie van Leeuwenhoek peered and examined dental plaque sampled from himself and others through his microscope. His sense of awe and his early appreciation of the diversity our microbial partners were evident. He named the microbes “Dierken”, meaning small lively objects (Gordon and Klaenhammer, 2011). Since then, people have been trying to discover the secrets of microorganisms. Over the next 200 years, with the advances in microscopy and other technologies, the understanding of microorganism has become more and more profound. The human oral cavity contains a number of different habitats, including the teeth, gingival sulcus, tongue, hard and soft palates, and tonsils, and acts the tube which connect the outside and the digestive tract and respiratory tract of human body, which provides the appropriate space for the colonization of microorganisms. The microorganisms found in the human oral cavity have been referred to as the oral microflora, oral microbiota, or oral microbiome (Dewhirst et al., 2010). Interaction of variable oral microorganisms helps human body against invasion of undesirable stimulation outside. However, imbalance of microbial flora contributes to oral diseases such as dental caries, periodontitis (Holt et al., 1988; Jorth et al., 2014; Liu et al., 2012; Philip et al., 2018; Wasfi et al., 2018; Costalonga and Herzberg, 2014), oral mucosal diseases (Saikaly, 2018) and systemic diseases, such as gastrointestinal and nervous systemic diseases (Jorth et al., 2014; Atarashi, 2017; Blod et al., 2017; Fardini et al., 2010; Kuczynski et al., 2012; Ling et al., 2015; Lirajunior and Boström, 2018; Peters et al., 2017; Plaza-Diaz et al., 2018; Reddy et al., 2018; Roszyk and Puszczewicz, 2017; Zarco et al., 2012). Oral microbiomes play an important role in the human microbial community and human health (Zarco et al., 2012).

## Oral microbiomes in human oral cavity

### Human oral microbiome database

Human oral microbiome database (HOMD) is the first curated description of a human-associated microbiome and provides tools for use in understanding the role of the microbiome in health and disease. The purpose of HOMD is to provide the scientific community with comprehensive information on the approximately 700 prokaryote species that are present in the human oral cavity. HOMD is based on a curated 16S rRNA gene-based provisional naming scheme. Over the past 20 years, the laboratory has sequenced over 600 16S RNA gene libraries and obtained over 35,000 clone sequences. The samples came from healthy subjects and subjects with over a dozen disease states such as caries, periodontal disease, endodontic infections and oral cancer. The HOMD links sequence data with phenotypic, phylogenetic, clinical and bibliographic information. The organization, integration and presentation of the HOMD data can use as a model for microbiome data from other human body sites such as gut, skin, vagina (Dewhirst et al., 2010; Costalonga and Herzberg, 2014; Blanton, 2012; Chen, 2010; Eren et al., 2014). Approximately 700 species listed in the HOMD, and 51 per cent of which are officially named, 13 per cent of which are not named (but cultivated) and 28 percent of which are known only as uncultivated phylotypes (Homepage, 2016). In the HOMD database, there are approximately 150 genera, 700 species. Genomes for 400 oral taxa and more than 1,300 strains of microorganisms are currently available on HOMD. For example, *streptococcus* is a genus that has higher abundance than many genera (Butler et al., 2017). In the HOMD, *Streptococcus* genus has 43 species, of which 26 are named, 9 are not named, 7 are dropped and 2 are Lost. Genomes for 30 oral taxa and 202 strains of *Streptococcus* are available on HOMD. In the HOMD, *Prevotella* genus has 53 species, of which genomes for 32 species and 67 strains are available on HOMD (All Human Oral Microbial Taxa, 2018). HOMD is based on the culture of microorganism. But limitation is quite a part of oral microorganisms of HOMD data aren’t able to be cultivated, of which as much as 20% to 60% has been estimated to be uncultivable (Human Microbiome Project Overview, 2017), because of the restriction of the culture conditions, the interaction of microbes and so on.

### NIH common fund Human Microbiome Project

**Figure. 1:**
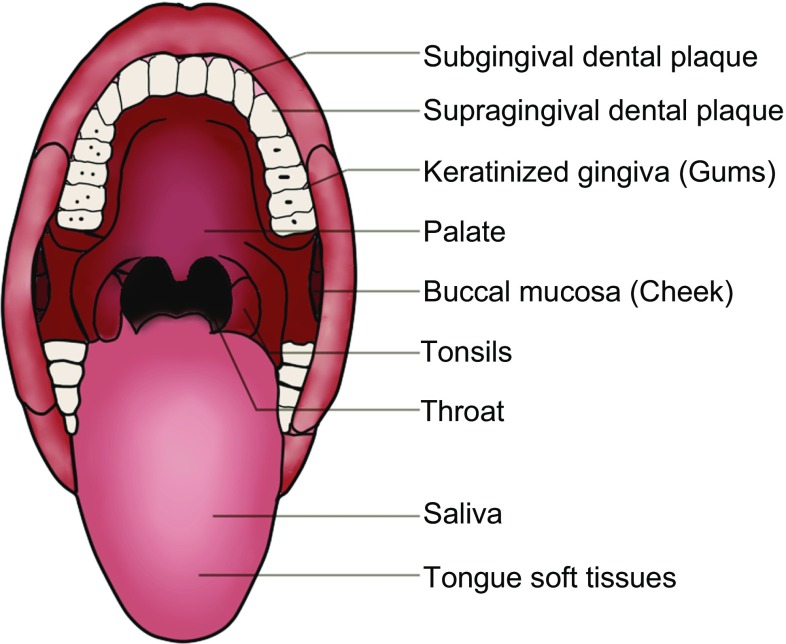
**Nine specimens were collected in the HMP population**. Saliva, buccal mucosa (cheek), keratinized gingiva (gums), palate, tonsils, throat and tongue soft tissues, and supra gingival and subgingival dental plaque (tooth biofilm above and below the gum)

The NIH common fund Human Microbiome Project (HMP) was established with the mission of generating research resources enabling comprehensive characterization of the human microbiota and analysis of their role in human health and disease at the end of 2007 (Peterson et al., 2009). A total of 4,788 specimens from 242 screened and phenotyped adults (129 males, 113 females) were available for HMP. Three hundred adult volunteers were enrolled at two clinical centers (Baylor College of Medicine, Houston, TX; Washington University, St. Louis, MO); these included equal numbers of 18- to 40-year-old men and women, of who in this study, 279 were sampled twice and 100 were sampled a third time over approximately 22 months (Human Microbiome Project, 2012). Based on a lengthy list of exclusion criteria, adult subjects lacking evidence of disease were recruited, and the researchers will refer to them as “healthy”, as defined by the consortium clinical sampling criteria. Women were sampled at 18 body habitats, men at 15 (excluding three vaginal sites), distributed among five major body areas, including the oral cavity, nasal cavity, vagina, intestinal tract and skin. Nine specimens were collected from the oral cavity and oropharynx, which is more than half of the habitats of habitats: saliva, buccal mucosa (cheek), keratinized gingiva (gums), palate, tonsils, throat and tongue soft tissues, and supragingival and subgingival dental plaque (Fig. 1). The bacteria of oral that have been sequenced accounted for 26% of all the body sites (Griffen et al., 2011; Ahn et al., 2011). Both overall community similarity and microbial co-occurrence and co-exclusion across the human microbiome grouped the 18 body habitats together into four clusters corresponding to the five target body areas. The oral cavity taxon, which was one of less dominant taxa, was suggested to be highly personalized. In the oral cavity, most habitats were dominated by Streptococcus, but these were followed in abundance by *Haemophilus* in the buccal mucosa, *Actinomyces* in the supragingival plaque, and *Prevotella* in the immediately adjacent (but low oxygen) subgingival plaque. The study also revealed relationship among microbial community membership and function. Furthermore, they indicated the variation of microbial carriage between subjects down to the species and strain level, and variation of carriage of microbial taxa while metabolic pathways remained stable within a healthy population (Ahn et al., 2011). Although HMP had done a lot of research on microbiology, due to the fact that 16S rRNA gene sequencing is limited to the segment of 16S rRNA and the identification of microorganisms should take into account the whole genome, this study had slight significance on the level of microbiology species. There were some approaches and other gene database to add more information to current public database (Eren et al., 2014; Costello et al., 2009; Ding and Schloss, 2014).

### Influence factors of oral microbiomes in healthy status

Healthy individual’s microbiology was associated with many factors.

1. Time: Costello et al., (2014) studied 27 sites in seven to nine healthy adults microbiota on four occasions. These results indicated that our microbiota was personalized, varied systematically across body habitats and time. The HMP Consortium reported the structure and function of the human microbiome in 300 healthy adults at 18 body sites over the course of 12–18 months. Over the course of the sampling period, the community types from sites within the oral cavity were more unstable (Anukam and Agbakoba, 2017). Ogawa et al., (2018) studied the salivary microbiota composition in samples from healthy and frail elderly individuals using 16S rRNA sequencing analysis. The study suggested that general frailty was associated with oral microbiota composition and formation.
2. Age: Anukam and Agbakoba (2017) collected oral samples from three randomly selected females aged 56, 28 and 8 years, extracted DNA and amplified 16S rRNA V4 region using custom-barcoded primers before sequencing with IlluminaMiSeq platform. The study revealed that bacteria with varying diversities colonized the subjects on females of different age groups. Since the life expectancy of human was prolonged with the progress of medical science, An et al., (2018) has studied the biology of aging and mechanisms of oral disease, hoping aiding the future study of geriatric health and suggesting that more clinical research should consider the important of age.
3. Diet: Lassalle et al., (2017) sampled saliva from three pairs of populations of hunter-gatherers and traditional farmers living in close proximity in the Philippines. The results suggested that major transitions in diet selected for different communities of commensals and likely played a role in the emergence of modern oral pathogens. Adler et al., (2013) studies indicated that the transition from hunter-gatherer to farming shifted the oral microbial community to a disease-associated configuration. Modern oral microbiotic ecosystems were markedly less diverse than historic populations, which might be contributing to chronic oral disease in postindustrial lifestyles. Brito et al., (2016) compared the mobile genes found in the microbiomes of 81 metropolitan North Americans with those of 172 agrarian Fiji islanders using a combination of single-cell genomics and metagenomics. They found large differences in mobile gene content between the Fijian and North American microbiomes. These results suggested that the abundance of some genes may reflect environmental selection. Many studies were involved in the ancient oral microbiome from well-preserved dental calculus to investigate the evolution of genomic, diseases and so on (Metcalf et al., 2014; Brown et al., 1976; Galvão-Moreira et al., 2017).
4. Extreme environment: Brown et al., (1976) measured the preflight and postflight monitoring of changes in microbial populations at various intraoral sites. Microbiologic assessments showed noteworthy elevations in counts of specific anaerobic components of the oral microflora, *Streptococci*, *Neisseria*, *Lactobacilli* and *Enteric bacilli*, which were believed to be diet related. The study suggested that the relative absence of intraoral changes that were hazardous to one’s health during spaceflight.
5. Other factors: HMP reported that there were strong associations between whether individuals had been breastfed as an infant, their gender, and their level of education with their community types at several body sites (Anukam and Agbakoba, 2017). Galvão-Moreira et al., (2017) studied 46 female and 24 male patients, aged 18–40 years, and counted both groups’ *streptococcus mutans*. The study suggested that there was a significant difference for *S. mutans* levels in both groups.

## Oral microbiomes and oral diseases

### Caries

Caries is the most common chronic infectious disease, taking bacteria as main pathogen and can lead to chronic and progressive destruction of dental hard tissue under many factors. Caries have a wide range and high incidence, which can occur at any age, from children to the old. And the early childhood caries is the most harmful and has become a prevalent public health problem among preschool children globally, which it has many factors influence the incidence of, including oral microbiome (Jenkinson and Lamont, 2005; Ma et al., 2015). Xu et al., (2014) detected changes in dental plaque microbial community profiles and oral behavioral habits during the transition from a caries-free to a caries state using polymerase chain reaction (PCR) denaturing gradient gel electrophoresis (DGGE) in 3-year-old caries-free children followed up for 12 months. They found for young Chinese children, the high frequency of eating sweets and eating sweets before sleeping are risk factors of caries onset, and decrease of microbial abundance occured 6 months before onset of caries. Xu et al. elucidated and monitored supragingival plaque bacterial diversity with second primary molar unerupted, observing differences in abundance for several microbial groups between caries and caries-free host populations, revealing distinctions between caries and caries-free microbiomes in terms of microbial community structure (Xu et al., 2014). Chen et al. used the human oral microbe identification microarray (HOMIM) to compare the bacterial profiles in saliva and supragingival plaque samples between children with severe early childhood caries (SECC) and caries-free children. They detected 379 bacterial species from all children and found several genera, including *Streptococcus*, *Porphyromonas* and *Actinomyces*, were strongly associated with SECC and could be potential biomarkers of dental caries in the primary dentition (Ma et al., 2015). Wang et al. characterized the oral microbiota by comparing and analysing saliva from 20 children with caries and 21 caries-free children of Han Chinese origin based on the single-molecule real-time DNA sequencing system. It was concluded that *Prevotella* spp., *Lactobacillus* spp., *Dialister* spp. and *Filifactor* spp. might be related to the pathogenesis and progression of dental caries (Wang et al., 2017). Agnello et al. utilized next-generation sequencing to analyze the plaque microbiome from Canadian first nations and Métis children, with and without SECC. They revealed that twenty-eight species-level operational taxonomic units were significantly different between the groups. *VeillonellaHOT 780* and *PorphyromonasHOT 284* were 4.6- and 9-fold higher, respectively, in the SECC group, and *Streptococcus gordonii* and *Streptococcus sanguinis* were 5- and 2-fold higher, respectively, in the caries-free group. Extremely high levels of *Streptococcus mutans*were detected in the SECC group (Agnello et al., 2017).

### Periodontal diseases

Periodontal diseases frequently occur in human mouth, and can be divided into two categories, gingival diseases and periodontitis. Periodontal diseases cause destruction of periodontium (tooth-supporting tissues such as gingiva and alveolar bone) and constitute a potential risk factor for certain systemic diseases (Agnello et al., 2017; Pihlstrom et al., 2005). Oral cavity is a natural microbial culture medium, in which periodontal tissue has complex anatomy and organizational structure, physical and chemical properties, which indeed provides good conditions for growth of microorganisms.

Topcuoglu et al. recruited 84 subjects, including generalized aggressive periodontitis (*n* = 29), generalized chronic periodontitis (*n* = 25), peri-implantitis (*n* = 14), localized aggressive periodontitis (*n* = 8), to sequenced 16S rRNA genes in 10 selected species. They finally found the red complex bacteria were the most prevalent with very high levels in all groups. *Fusobacterium nucleatum* (*F. nucleatum*)was detected in all samples at high levels. The green and blue complex bacteria were less prevalent compared with red and orange complex, except *Aggregatibacter actinomycetemcomitas* was detected in all localized aggressive periodontitis groups (Hajishengallis, 2015; Topcuoglu and Kulekci, 2015). Pozhitkov et al. extracted DNA and amplified 16S rRNA genes of the oral microbiome in subjects with periodontitis of 16 systemically healthy white adults with clinical signs of one of the following oral conditions (periodontitis, established caries, edentulism and oral health) showing the greatest diversity harboring 29 bacterial species at significantly higher abundance compared to subjects with the other assessed conditions (Pozhitkov et al., 2015). Some studies suggested microbiome abundances were significantly different between shallow and deep sites of teeth (Ge et al., 2013). Tsai et al. found high microbial diversity, with an average of 774 classified phylotypes per sample and a total of six bacterial phyla across all samples (Tsai et al., 2016).

### Mucosal diseases

Oral leukoplakia (OLK), oral lichen planus (OLP) and systemic lupus erythematosus (SLE) are common diseases of oral mucosa or specific manifestation of systematic diseases in oral mucosa, which draw a lot of attention of the public. OLK is defined as a white oral lesion not related to another disease process and the lesion is largely asymptomatic (Bewley and Farwell, 2017). OLP is one of the most common chronic inflammatory autoimmune diseases (Reichart et al., 2016). OLP with long-term erosion has the risk of turning to cancer. SLE is a chronic autoimmune disease with a heterogeneous course and systemic involvement. It is the result of a complex pathogenic pathway that culminates in autoantibody formation (Yeoh et al., 2018). Several studies have demonstrated that bacteria play an important role in these mucosal diseases (Hu et al., 2016).

Researchers in Peking University School and Hospital of Stomatology collected saliva and extracted DNA of 10 patients with OLK, and 19 patients HCs enrolled in this study from PKUSS, Beijing. They used the IlluminaMiSeq to prosequence of 16S rRNA and compared with those for healthy controls (HCs), and the data showed *Haemophilus* was much more abundant in the OLK group (1.51%) than in the HC (0.34%) groups which demonstrated OLK might be associated with changes in the salivary microbiota (Hu et al., 2016). By comparing DNA extracted from swads of OLK (*n* = 36) and healthy controls (*n* = 32), Amer et al. obtained increased abundance of *Fusobacteria* and reduced levels of *Firmicutes* in patients with OLK. Moreover, severe dysplasia was associated with elevated levels of *Leptotrichia* spp. and *Campylobacter concisus* (Amer et al., 2017).

Lichen planus is a common chronic mucocutaneous inflammatory. The prevalence of OLP ranges from 0.1%–4% in the general population (Lodi et al., 2005). Wang et al. utilized MiSeq sequencing of 16S rRNA gene amplicons to identify complex oral microbiota associated with OLP from saliva samples of two subtypes (reticular and erosive) of OLP patients and healthy controls, and observed evident variations in abundance for several taxonomic groups in OLP (Wang et al., 2016).

Corrêa evaluated 52 patients with SLE and 52 subjects without SLE (control), and amplified the V4 region of 16S rRNA gene from subgingival dental plaque DNA extracts. Compositions of oral microbiota in SLE individuals were different (Corrêa et al., 2017).

### Oral cancer

Several factors take effect on the occurrence and development of oral cancer, such as gene, bacteria, body status and so on. Emerging evidence suggests a link between microbiome and oral cancer. Squamous cell carcinoma is that the most frequently occurring malignancy of the oral cavity and adjacent sites, representing over 90% of all cancers (Gholizadeh et al., 2016).

**Figure 2:**
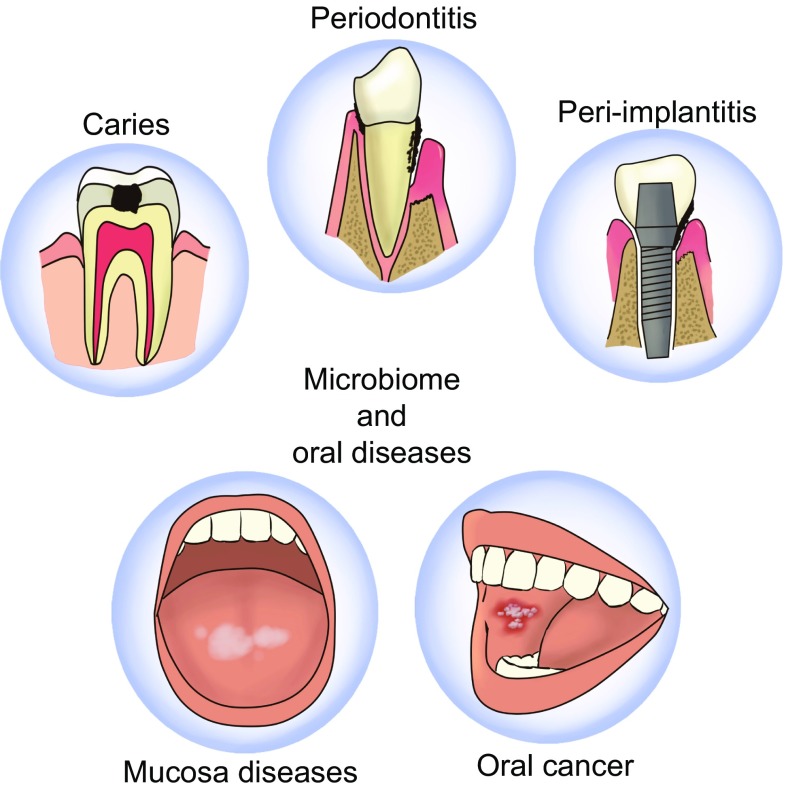
**Oral microbiome and oral diseases**. Various kinds and various numbers of bacteria have been found in people with different oral diseases such as dental caries, peridontal diseases, mucosal diseases (e.g., lichen planus, leukoplakia), oral cancer and peri-implantitis

Nagy collected biofilm samples obtained from the central surface of the lesions in 21 patients and contiguous healthy mucosa, and cultured *in vitro*. Finally they achieved the conclusion that human oral carcinoma surface biofilms harbour significantly increased numbers of aerobes and anaerobes as compared with the healthy mucosal surface of the same patient (Nagy et al., 1998). Lee et al. investigated microbiota differences between normal individuals, epithelial precursor lesion patients and cancer patients by using next-generation sequencing. They revealed that abundance of *Bacillus*, *Enterococcus*, *Parvimonas*, *Peptostreptococcus* and *Slackia* showed significant difference between epithelial precursor lesion and cancer patients and correlated with classification into 2 clusters (Lee et al., 2017). Yang et al. used 16S rRNA amplicon sequencing to study the composition of oral microorganisms in oral squamous cell carcinoma (OSCC) patients and found potential association of oral microbiomes with mutational changes in OSCC (Yang et al., 2018) (Fig. 2).

### Peri-implantitis

Dental implants are commonly used to replace missing teeth. Implant therapy was introduced to dentistry 50 years ago and has become one of the routine procedures for replacing the missing tooth. However, when people enjoy the aesthetics and fine function, they meanwhile obtain some complications like peri-implantitis. Peri-implantitis is an infectious disease characterized by inflammation of the tissues surrounding the implant, bleeding on probing with or without suppuration, and bone loss (de Araújo et al., 2017). There are some proved differences in oral microbiomes between peri-implantitis patients and healthy individuals (Zheng et al., 2015). Lafaurie et al. found that peri-implantitis represented a heterogeneous mixed infection that includes periodontopathic microorganisms (Lafaurie et al., 2017). Zheng et al. analyzed the microbial characteristics of oral plaque from peri-implant pockets or sulci of healthy implants (*n* = 10), peri-implant mucositis (*n* = 8) and peri-implantitis (*n* = 6) sites and compared the data, which revealed that *Eubacterium minutum* was correlated with *Prevotella intermedia* in peri-implantitis sites, suggesting the association of *Eubacterium* with peri-implantitis. These findings indicated that periodontal pathogens might be closely related to peri-implantitis (Zheng et al., 2015).

## Oral microbiomes and whole-body systematic diseases

**Figure 3:**
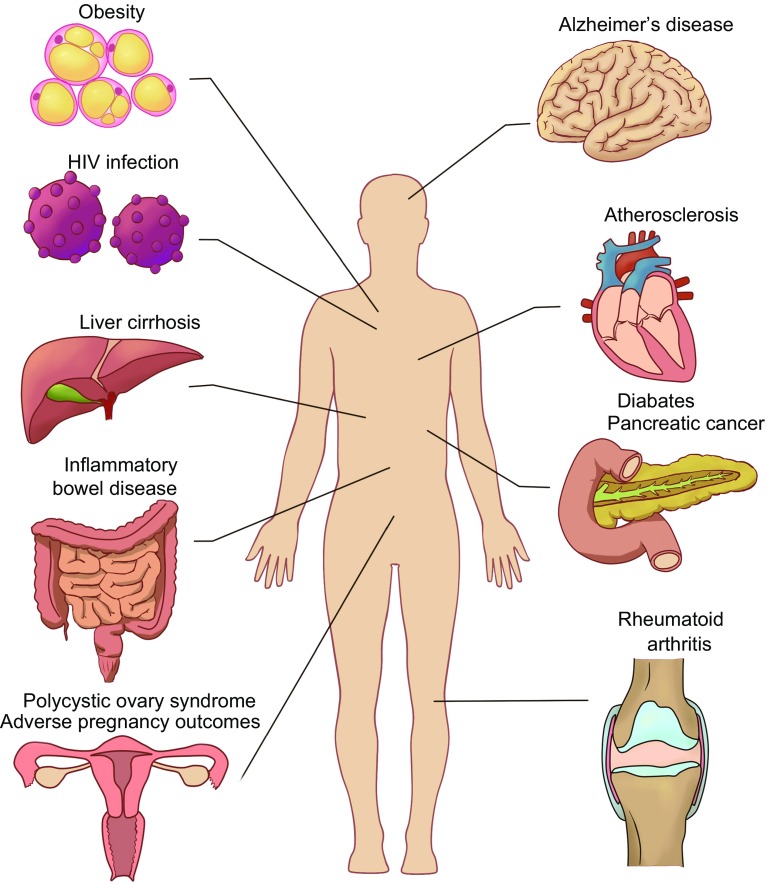
**Oral microbiomes and whole-body systematic diseases**. Oral microbial dysbiosis contributes to variable systemic diseases processing including gastrointestinal system diseases like inflammatory bowel disease, liver cirrhosis, pancreatic cancer, nervous system diseases like Alzheimer’s disease, endocrine system diseases like diabetes, adverse pregnancy outcomes, obesity and polycystic ovary syndrome, immune system diseases like rheumatoid arthritis and HIV infection, and cardiovascular system diseases like atherosclerosis

The oral cavity is the initial point of entry to the digestive and respiratory tract. Over 700 bacterial species may be found in the oral cavity of humans (Paster et al., 2006). Oral microbial dysbiosis is linked to oral inflammation and may contribute to systemic conditions through bacteremia (Han and Wang, 2013) (Fig. 3).

### Gastrointestinal system diseases

More and more gastrointestinal system diseases are proved to be associated with oral microbiomes. Inflammatory bowel disease (IBD) is one of the earliest to be found. Nowadays, there’re more convincing evidences for correlations between liver cirrhosis, gastrointestinal cancers and oral microbiomes.

#### Inflammatory bowel disease

Inflammatory bowel disease (IBD) includes a spectrum of diseases from ulcerative colitis (UC) to Crohn’s disease (CD) (Khor et al., 2011). UC is characterized by continuous, diffuse and superficial inflammation of the colon (Ford et al., 2013). CD is a chronic inflammatory disease that may affect different regions of the digestive tract, from mouth to the anus, although it most commonly affects the colon and terminal ileum (Baumgart et al., 2007).

The pathogenesis of IBD is currently thought to involve an inappropriate and persistent inflammatory response to commensal gut microbiomes in genetically susceptible individuals. IBD has been explained as the result of complex interactions among genetic, immunological, microbiological and environmental factors (Gentschew and Ferguson, 2012; Kaistha and Levine, 2014; Neuman and Nanau, 2012). Loss of antigen tolerance stimulates differentiation of T-helper (Th) cells and production of proinflammatory cytokines (e.g., tumor necrosis factor α and IL(interleukins)-1β, IL- 6, IL-12 and IL-23) and chemokines (Baumgart et al., 2007). Inflammatory cells attracted by interleukins and chemokines then release non-specific inflammatory substances (cellular metabolites like polyunsaturated omega-6 fatty acid arachidonic acid, proteases, platelet activation factor and free radicals) that bring about intestinal damages (Radford-Smith and Pandeya, 2006). Intestinal fungi are an important component of the microbiota, and recent studies have unveiled their potential in modulating host immune homeostasis and inflammatory disease. Leonardi et al. identified CX3CR1^+^ (CX3C chemokine receptor 1) mononuclear phagocytes (MNPs) as being essential for the initiation of innate and adaptive immune responses to intestinal fungi. CX3CR1^+^ MNPs express antifungal receptors and activate antifungal responses in a Syk-dependent manner, unraveling a role of CX3CR1^+^ MNPs in mediating interactions between intestinal mycobiota and host immunity at steady state and during inflammatory disease (Leonardi et al., 2018).

Physiologically, the intestine has developed several strategies to resist colonization by non-native bacteria and control the expansion of pathobionts that have the potential to cause pathology. Intestinal colonization by bacteria from the oral cavity has been suggested to be extensively involved in inflammatory diseases (Pickard et al., 2017; Caballero and Pamer, 2015).

Patients suffering from IBD often show various oral symptoms such as aphthous stomatitis, oral ulcer, dry mouth and pyostomatitis vegetans are frequently observed in IBD patients (Jose and Heyman, 2008; Veloso, 2011), suggesting potential association of oral microbiota with such manifestations. However, there is still very limited information about the oral microbiota from IBD patients (Said et al., 2014). One recent study showed that *Bacteroidetes* was significantly increased with a concurrent decrease in *Proteobacteria* in the salivary microbiota of IBD patients. The dominant genera, *Streptococcus*, *Prevotella*, *Neisseria*, *Haemophilus*, *Veillonella* and *Gemella*, were found to largely contribute to dysbiosis observed in the salivary microbiota of IBD patients (Said et al., 2014). This study also reported that the observed dysbiosis was strongly associated with elevated inflammatory response of several cytokines with depleted lysozyme in the saliva of IBD patients, some of which showed a strong correlation with the relative abundance of certain bacterial species. For example, there’s a strong correlation between lysozyme and IL-1β levels and the relative abundance of *Streptococcus*, *Prevotella*, *Haemophilus* and *Veillonella*. Another study reported that dysbiosis observed in murine models of colitis is associated with composition change of bacteria present in the oral cavity and in saliva (Said et al., 2014; Lucas López et al., 2017).

One more recently study by Atarashi et al. showed that strains of *Klebsiella* spp. from the salivary microbiota colonize in the gut and can potently induce chronic intestinal inflammation (Atarashi et al., 2017). Strains of *Klebsiella* spp. isolated from the salivary microbiota are strong inducers of Th1 cells when colonizing in gut by using gnotobiotic techniques. These *Klebsiella* strains are resistant to multiple antibiotics, tend to colonize when intestinal microbiota is dysbiotic, and elicit severe gut inflammation in the context of a genetically susceptible host. Oral cavity may serve as a reservoir for potential intestinal pathobionts that can exacerbate intestinal disease.

There is clearly a need for more studies on the oral microbiome from IBD patients, and there are also a number of questions that need to be solved such as possible differences between CD and UC, and on the influence of other factors such as age, diet and medication on microbial dysbiosis associated with IBD (Lucas López et al., 2017; Atarashi et al., 2017).

#### Other gastrointestinal system diseases

Liver cirrhosis occurs as a consequence of many chronic liver diseases that are prevalent worldwide. Qin et al. characterized gut microbiome in liver cirrhosis, building a reference gene set for the cohort (Qin et al., 2014). 75,245 genes that differ in abundance between groups can be grouped into 66 clusters representing cognate bacterial species; 28 are enriched in patients and 38 in control individuals. 54% of the patient-enriched, taxonomically assigned species are of buccal origin, suggesting an invasion of gut from mouth in liver cirrhosis. Biomarkers specific to liver cirrhosis at gene and function levels are revealed by comparison with those for type 2 diabetes and IBD. On the basis of only 15 biomarkers, a highly accurate patient discrimination index is created and validated on an independent cohort. Thus microbiota-targeted biomarkers may be a powerful tool for diagnosis of different diseases.

Gastrointestinal cancer risk increases in individuals with periodontal disease or tooth loss, conditions caused by oral bacteria (Meurman, 2010; Rogers and Fox, 2004). Oral bacteria may activate alcohol and smoking-related carcinogens locally or act systemically, through chronic inflammation (Ahn et al., 2012). Pancreatic cancer is a kind of highly lethal gastrointestinal cancer. A history of periodontal disease and the presence of circulating antibodies to selected oral pathogens have been associated with increased risk of pancreatic cancer. Fan et al. examined the relationship of oral microbiota with subsequent risk of pancreatic cancer in a large nested case-control study (Fan et al., 2016). In the direct assessment of genomic-based microbiome in oral samples, carriage of the oral pathogens *Porphyromonas gingivalis* and *Aggregatibacter actinomycetemcomitans* was associated with increased risk of pancreatic cancer. *Leptotrichia* genus was associated with decreased risk of pancreatic cancer. These oral bacteria may additionally serve as readily accessible, non-invasive biomarkers for subsequent pancreatic cancer risk, which helps to identify people at high risk for this disease. Furthermore, targeted prophylactic therapies may be developed to combat periodontal pathogens and decrease risk for pancreatic cancer (Fan et al., 2016).

### Nervous system diseases

Connections between nervous system diseases and oral microbiomes have been proved. It’s inspiring to cognize nervous system diseases on a new perspective. Alzheimer’s disease (AD) is a typical example.

AD is the most common example of dementia causing around 60%–80% of all cases, characterized by cognitive deficit and has a complex, multifactorial etiology (Gaugler et al., 2016). Miklossy et al. highlighted involvement of several types of spirochetes in AD including oral and intestinal (Miklossy, 1993). Riviere et al. found oral anaerobes (phyla Treponema) in brain samples by PCR technology and species-specific antibodies (Riviere et al., 2002). Treponema were also detected using antibodies in 15 out of 16 AD brains compared with 6 of 18 controls, suggesting that certain bacterial phyla are more closely associated with AD, since they were not as heavily represented in the non-AD samples. This is consistent with evidence of lipopolysaccharide from oral anaerobe Porphyromonas gingivalis in brains of AD patients and not controls (Poole et al., 2013).

The association between raised TNF-α and AD is well established. Kamer et al. used standard ELISA technique with antibodies to detect TNF-α and looked for serum antibodies for periodontal bacteria *Actinobacillus actinomycetemcomitans*, *Tannerella forsythia* and *Porphyromonas gingivalis*. Levels of TNF-α and antibodies for oral bacteria were higher in AD patients compared to controls and the presence of serum antibodies for these bacteria carried an odds ratio of 6.1 for AD. This could be used as a diagnostic tool (Kamer et al., 2009). Furthermore, a longitudinal study has explored the potential for using oral bacteria as a predictive tool. 158 people from biologically resilient adults in neurological studies research program at the University of Kentucky were all cognitively normal at baseline. Raised baseline antibody levels, specific for the oral anaerobes *F. nucleatum* and *Prevotellaintermedia*, correlated with cognitive deficits in subjects 10 years later (Sparks Stein et al., 2012).

### Endocrine system diseases

Processing and prognosis of endocrine system diseases are closely related to individual internal environment. Oral microbiomes influence and can be influenced by individual internal environment, which enlighten us to find correlations between endocrine system diseases and oral microbiomes. Diabetes, adverse pregnancy outcomes (APOs) and obesity have been proved to be associated with oral microbiomes.

#### Diabetes

Diabetes mellitus is characterized by hyperglycemia, inflammation and high oxidative stress, which can lead to systemic complications. There is a bidirectional relationship between periodontal disease and diabetes. Microbiome plays a key role in homeostasis and affects several pathologic processes, including diabetes (Ussar et al., 2016).

Diabetes is a risk factor for periodontitis and increases disease severity. In type I diabetics, an increase in the severity of periodontal diseases has been shown across most age ranges. Age itself has been shown to be a risk factor for periodontitis, and is likely to be a confounder (Cullinan et al., 2001; Rylander et al., 1986; Cianciola et al., 1982; Thorstensson and Hugoson, 1993). Type II diabetes has also been shown to be a risk factor for periodontal diseases. A study of association between diabetic status and periodontal conditions in 1,342 individuals showed increased risk for periodontitis (Emrich et al., 1991).

Casarin et al. observed significant differences in subgingivalmicrobiota between type-II diabetes and nondiabetic subjects such as higher percentage of *TM7*, *Aggregatibacter*, *Neisseria*, *Gemella*, *Eikenella*, *Selenomonas*, *Actinomyces*, *Capnocytophaga*, *Fusobacterium*, *Veillonella* and *Streptococcus* genera (Casarin et al., 2013).

Xiao et al. provide a mechanistic basis for better understanding how diabetes increase risk and severity of tooth loss (Xiao et al., 2017). Diabetes causes a shift in oral bacterial composition and, by transfer to germ-free mice, that the oral microbiota of diabetic mice is more pathogenic. Furthermore, treatment with IL-17 antibody decreases the pathogenicity of the oral microbiota in diabetic mice; when transferred to recipient germ-free mice, oral microbiota from IL-17-treated donors induced reduced neutrophil recruitment, reduced IL-6 and RANKL and less bone resorption. Diabetes-enhanced IL-17 alters the oral micro biota and renders it more pathogenic.

#### Adverse pregnancy outcomes

Adverse pregnancy outcomes (APOs) have been found to be associated with oral microbiome changes. Madianos et al., (2013) found that APOs mothers had significantly higher levels of *Bacteroides forsythus* and *Campylobacter rectus*. Then, *F. nucleatum*, which is associated with periodontal disease, is also found to be associated with APOs. *F. nucleatum* may be transmitted hematogenously to the placenta and cause adverse pregnancy outcomes (Han et al., 2004; Han et al., 2010). The elicited systemic inflammatory response may exacerbate local inflammatory responses at the foeto-placental unit and further increase the risk for APOs (Madianos et al., 2013).

#### Other endocrine system diseases

Obesity has also been found to be associated with oral microbiome. As the inflammatory nature of obesity is widely recognized, Goodson et al. found composition of salivary bacteria changes in overweight women. Bacterial species could serve as biological indicators of developing overweight condition. Oral bacteria may participate in the pathology that leads to obesity (Goodson et al., 2009).

Polycystic ovary syndrome (PCOS) is a common female endocrine condition of unclear etiology characterized by hyperandrogenism, amenorrhoea and polycystic ovarian morphology, often complicated by infertility, obesity, insulin resistance and low-grade inflammation. The gut microbiome is known to contribute to several of these conditions. Recently, an association between stool and saliva microbiome community profiles was shown (Lindheim et al., 2016). PCOS patients showed a decrease in bacteria from the phylum *Actinobacteria* and a borderline significant shift in bacterial community composition.

### Immune system diseases

Oral microbiomes are strongly related to human immune system functions, thus are correlated with human immune system diseases like rheumatoid arthritis (RA), and make difference on multi-system diseases performances in immune system, such as human immunodeficiency virus (HIV) infection.

#### Rheumatoid arthritis

RA is an autoimmune disorder, associated with increased mortality owing to cardiovascular and other systemic complications. However, etiology of RA remains elusive. Although studies on genetic predisposition to RA have implicated genes such as HLA-DRB1, TNFAIP3, PTPN22 and PADI4, environmental factors have also been shown to contribute to disease pathogenesis (McInnes and Schett, 2011; Raychaudhuri et al., 2012; Okada et al., 2014; McInnes and Schett, 2007; Viatte et al., 2013).

Microbial triggers have been implicated in RA (Zhang et al., 2015). Concordance was observed between the gut and oral microbiomes, suggesting overlap in the abundance and function of species at different body sites. Dysbiosis was detected in the gut and oral microbiomes of RA patients, but it was partially resolved after RA treatment. Alterations in the gut, dental or saliva microbiome distinguished individuals with RA from healthy controls, were correlated with clinical measures and could be used to stratify individuals on the basis of their response to therapy. In particular, *Haemophilus* spp. were depleted in individuals with RA at all three sites and negatively correlated with levels of serum autoantibodies, whereas *Lactobacillus salivarius* was over-represented in individuals with RA at all 3 sites and was present in increased amounts in cases of very active RA. Functionally, the redox environment, transport and metabolism of iron, sulfur, zinc and arginine were altered in the microbiota of individuals with RA. It suggests potential for using microbiome composition for prognosis and diagnosis.

#### Human immunodeficiency virus (HIV) infection

HIV infection is associated with a range of oral conditions, and increased numbers of disease-associated microbial species have previously been found in HIV-positive subjects. Elevated viremia in untreated patients is associated with significantly higher proportions of potentially pathogenic *Veillonella*, *Prevotella*, *Megasphaera* and *Campylobacter* species than in healthy controls (Dang et al., 2012). Another study reported that microbial diversity in the oral cavity of HIV-infected individuals was lower than healthy controls, and this diversity was further reduced following ART treatment (Li et al., 2014). No significant differences between well-controlled HIV-positive patients and HIV-negative controls, suggesting that well-controlled HIV-positive patients essentially harbor similar oral flora compared to patients without HIV.

These evidences suggest that there is a shift in the oral microbiome and these changes might be associated with HIV infection and/or HIV-treatment and other oral manifestations associated with disease (Heron and Elahi, 2017).

### Cardiovascular system diseases

Correlations between cardiovascular system diseases aren’t strong enough for now, but researchers did proved some potential connections between atherosclerosis and oral microbiomes.

Atherosclerosis is characterized by accumulation of cholesterol and recruitment of macrophages to the arterial wall. It can thus be considered both a metabolic and an inflammatory disease (Hansson, 2005; Koren et al., 2011). By 16S rRNA sequencing, Koren et al. identified *Chryseomonas*, *Veillonella* and *Streptococcus* in the majority of atherosclerotic patients’ oral mocrobiomes (Koren et al., 2011). Moreover, several additional bacterial phylotypes were common to the atherosclerotic plaque and oral or gut samples within the same individual. Interestingly, several bacterial taxa in the oral cavity and the gut correlated with plasma cholesterol levels. Bacteria from the oral cavity, and perhaps even gut, may correlate with disease markers of atherosclerosis (Koren et al., 2011; Libby et al., 2002).

## Conclusion

The use of recently developed molecular methods has greatly expanded our knowledge of the composition and function of the oral microbiome in health and disease. Interaction and balance of a variety of oral microorganisms help human body against invasion of the undesirable stimulation outside. However, imbalance of microbial flora contributes to oral and whole-body systematic diseases. Oral microbiomes play an important role in human microbial community and human health status. Studies in oral microbiomes and their interactions with whole-body microbiomes in variable body sites and variable health condition are critical in our cognition of human body and how to make effect on human health improvement.
